# Orphan devices: yesterday is history; tomorrow is mystery: towards a European orphan device directive?

**DOI:** 10.1186/s13023-016-0393-3

**Published:** 2016-03-03

**Authors:** Marc M Dooms

**Affiliations:** University Hospitals Leuven, Leuven, Belgium

**Keywords:** History of medicine, Rare diseases, Orphan drugs, Custom-made medical devices, Humanitarian-use devices

## Abstract

**Background:**

Regulatory and economic frameworks stimulated the research and development of orphan drugs, but very little has been done for devices necessary for the in-vivo diagnosis, prevention and treatment of life-threatening conditions with a low prevalence/incidence.

**Discussion:**

A general public consultation in Europe has shown a positive attitude towards an “orphan device” directive. The United States of America have a Humanitarian Use Device exemption, but Europe is still waiting for such a stimulating framework. Post-marketing surveillance (“materio-vigilance”) will be necessary for follow-up, patient-reported outcome measures (quality of life versus survival) needed and off-label use data available for patient-safety reasons.

**Summary:**

The marketing period for devices is shorter than for medicinal products. Incentives are necessary to stimulate research and development of such “orphan devices” especially when surgical intervention is the only option.

Orphan devices are medical devices intended for the in-vivo diagnosis, prevention or treatment of a very rare life-threatening or chronically debilitating condition. Several institutions (for example, the United States Food and Drug Administration (FDA), the European Medicines Agency (EMA), and the Australian Therapeutic Goods Administration (TGA)) have put in place regulatory and economic frameworks to facilitate the development of orphan drugs (118 authorized by EMA to date), but much needs to be done for similar medical devices, especially in Europe. Do we need an EU-directive for orphan devices?

## Historical background

During the twentieth century, several breakthroughs in surgery were achieved [1]. For example, Alfred Blalock (1899–1964) developed a surgical procedure in 1944 to relieve the cyanosis in the Blue Baby Syndrome, a kidney was transplanted between identical twins in 1954, and a liver transplantation was performed in 1963 by Thomas Starzl (°1926). Numerous new techniques were developed such as direct blood transfusion by George Washington Crile (1864–1943) in 1905, amniocentesis in 1952 by Douglas Bevis (1919–1994), and diagnostic ultrasound by Ian Donald (1910–1987) in 1958.

New “innovative” instruments were also devised to save the lives of patients with low-prevalence diseases. But without any incentive it took the developers years in the past to get these life-saving instruments to the market. I will describe a few such cases here.

1. The negative pressure ventilator (“Iron Lung”)

**Fig. 1:**
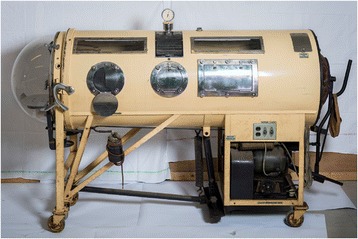
The negative pressure ventilator (“Iron Lung”)

A negative pressure ventilator (“Iron Lung”) is a medical ventilator that enables a patient to breathe when spontaneous breathing control has been lost or exceeds the patient’s ability. This device was invented by Philip Drinker (1894–1972) and Louis Agassiz Shaw (1886–1940) at the Harvard School of Public Health after an idea of John Dalziel in 1832. The first clinical use (12 October 1928) was with an 8-year-old girl with respiratory failure due to poliomyelitis. The patient was placed in a cylindrical steel drum. A door allowing the head and neck to remain free (left at Fig. 1) was then closed, forming a sealed, air-tight compartment enclosing the rest of the body. Pumps (lower right at Fig. 1) periodically decrease and increase the air pressure within the chamber and particularly on the chest. When the pressure is less than that within the lungs, the lungs expand and atmospheric pressure pushes air in. When the pressure goes above that within the lungs, the air is expelled [2]. This device is also used for Ondine’s curse and other rare conditions in which failure of the medullary respiratory centers in the brain results in patients having no autonomic control of breathing. Poliomyelitis vaccinations (Jonas Salk 1952) have virtually eradicated new cases of poliomyelitis, and the iron lung has virtually disappeared from modern medicine.

2. Stereotactic brain surgery

**Fig. 2:**
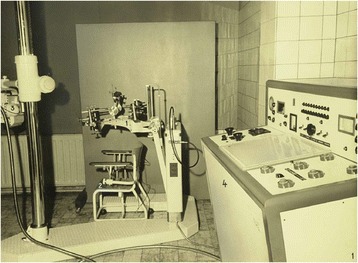
Stereotactic brain surgery

Stereotactic brain surgery was devised as a minimally invasive form of surgical intervention in the human brain that uses a three-dimensional coordinate system to locate small targets inside the brain and to perform some action such as injection and/or stimulation. The patient, under local anesthesia, sat on a chair (Fig. 2) and the mechanical head-holding clamps and bars kept the head of the patient in a fixed position in reference to the coordinate system. The stereotactic method was first developed in 1908 at the University College London Hospital by a British neurosurgeon, Victor Horsley (1857–1916), and the physiologist Robert H Clarke. This technique was the start of neuro-stimulation and evolved also to Deep Brain Stimulation (DBS), and neurosurgical methods for patients with rare forms of Parkinson’s disease, obsessive compulsive disorders, hyperkinesia, dystonia and convulsive diseases. In 2007, the EMA approved Gliolan (5-aminolevulinic acid oral solution) as an orphan drug to localize the tumor during brain surgery: when illuminated under blue light, the tumor cells glow an intense red when Gliolan is taken orally, while the normal brain tissue appears blue.

3. Incubator

**Fig. 3:**
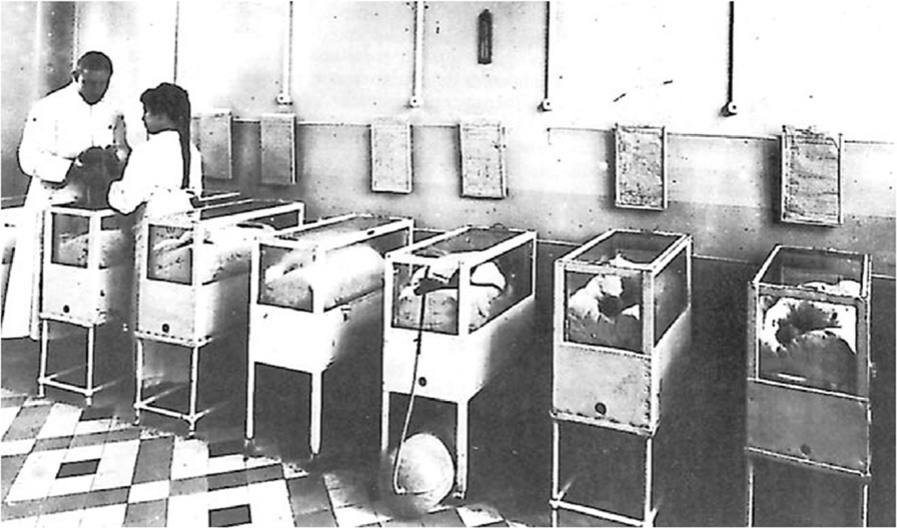
Incubator

An incubator (“*isolette*”) is an apparatus used to maintain environmental conditions suitable for a neonate: a sufficiently humidified and heated environment. It was used in preterm births or some ill full-term babies sometimes with a rare disorder. Stephane Tarnier (1828–1897) is generally considered to have been the father of the incubator, which he developed in a Paris maternity ward [3]. Oxygen was given freely (Fig. 3) until the end of the 1950’s when it was shown that the high concentrations reached inside incubators sometimes caused babies to go blind. Early incubators were even shown at commercial exhibitions with babies inside (*Exposition Universelle de Bruxelles 1897*: “*Une visite aux couveuses d’enfants ne s’oublie jamais*”). Since the Second World War, special-care baby units were established all over the world to provide neonatal care. The EMA authorized several orphan drugs to be used in neonatology such as Pedea (ibuprofen) for the treatment of hemodynamically significant patent ductus arteriosus in preterm newborn infants in 2004 and Peyona (caffeine) for the treatment of primary apnea in premature newborns in 2009.

4. Dialyzer

**Fig. 4:**
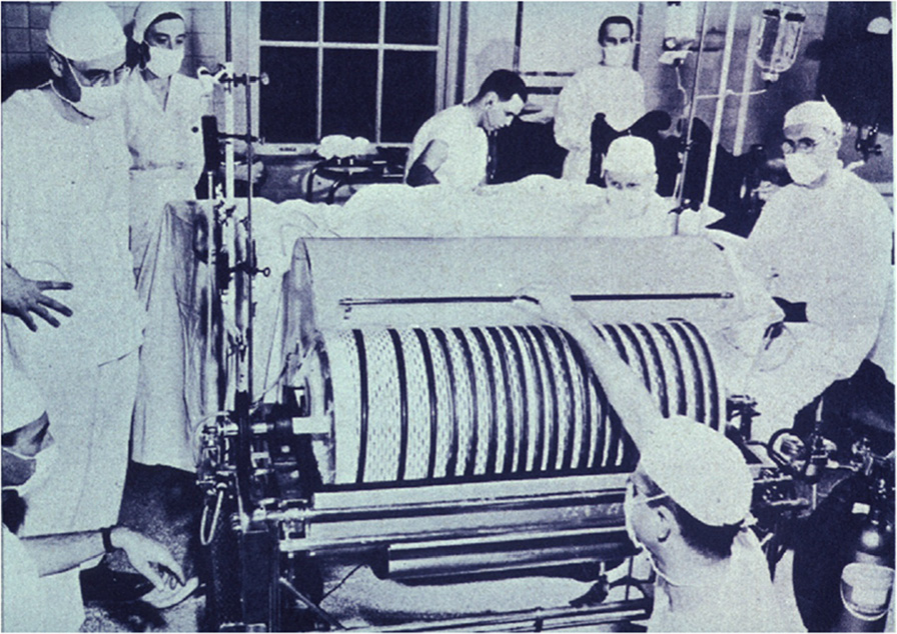
Dialyzer

The Dutch physician Willem Johan Kolff, (1911–2009) constructed the first working dialyzer in 1943 inspired by a scientific article from 1913 of John Abel. Nils Alwall (1904–1986) modified his construction later by enclosing it inside a stainless steel canister [4]. He treated his first patient, suffering from acute renal failure, on September 3, 1946. The blood contained in the 30 windings of cellophane tube which was plainly visible (Fig. 4). The cylinder was provided with ridges. Over the years this large machine was miniaturized (“wearable artificial kidney” or hemofiltration) and is now used in the treatment of some patients with end-stage renal disease. Some patients perform peritoneal dialysis at home. Procysbi (cysteamine bitarate caps), a controlled release form of Cystagon, was authorized in 2013 by EMA to delay the development of renal failure in patients with nephropathic cystinosis.

5. Orthopedic traction table

**Fig. 5:**
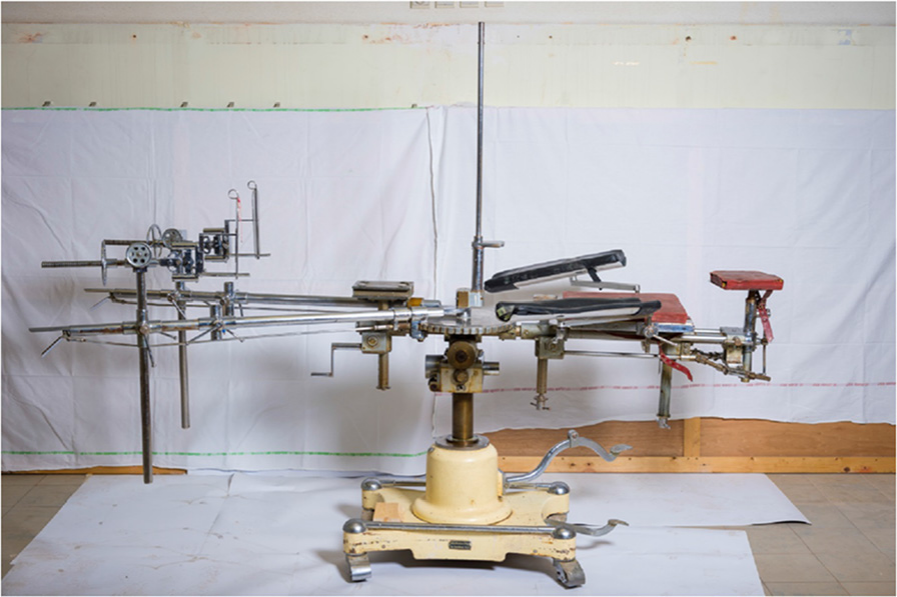
Orthopedic Traction Table

Orthopedic surgery was originally restricted to the correcting of musculoskeletal deformities in children such as clubfoot and curvatures of the spine. Spine extension methods are being used since antiquity and a traction table was described by Hippocrates (460–370 BC). The internal fixation of fractures was made possible with the development of orthopedic traction tables [5] (see Fig. 5). The bones of patients with *Osteogenesis imperfecta* (also known as the Brittle Bone Disease or the Lobstein Syndrome) were surgically corrected and rods were placed inside the bones to enable them to learn to walk. Dr. E A Miller described the process in 1959.

Operating theaters now have a large collection of sterile medical material to be implanted during surgical interventions such as neuro-stimulators, prostheses, heart valves, stents, osteosynthesis material and pacemakers. All this material is evaluated by “notified bodies” in every EU-Member State (Regulation 93/42/EEC from 14 JUNE 1993 concerning medical devices and Regulation 90/385/EEC concerning implantable active medical devices) and assigned a CE (Conformité Européenne) marking when approved. Different systems have also been used to evaluate post-marketing the efficacy and safety (“materio-vigilance”, Eudamed) of such devices. The use of a medical device outside the population or purpose for which the safety and effectiveness profile has been evaluated (off-label use) is quite common with low-prevalence diseases (mainly in children). To encourage the research into, and development of, medical material (“orphan devices”) for the in-vivo diagnosis, prevention and treatment of rare diseases we do need incentives such as a centralized European procedure and protocol assistance. This is of particular urgency because the marketing period for devices is shorter than for medicinal products: the risk on obtaining no return on investment for R & D on devices is real. The absence of incentives will become more important as we continue to enter the era of personalized medicine with the capabilities of bio-sensors, micro-fluidic tissue/organs on a micro-chip, artificial organs and diagnostic imaging instruments.

### Actual opinions within EU member states

A general public consultation of the European Commission (2007) has been launched to find out if the EU should have an orphan regulation on medical devices and diagnostics [6] (Question 9). Hundreds of responders (patients and their families, national and international (patient) organizations, national authorities, commercial organizations and companies, universities and experts, reference centers and researchers) were in favor of such a regulation except the following six:

*The *Association Internationale de la Mutualité* thought there is neither enough information nor evidence to justify it.
*The European Social Insurance Platform mentioned that medical devices already on the market were not specific to “rare disease”.
*The UK National Health Service stated that they did not believe that there were sufficient problems in the development and commercial marketing of devices to justify the administrative effort and special privileges for orphan regulations.
*The Ministry of Health, The Elderly and Community Care in Malta felt that such a regulation would neither be necessary nor beneficial and that the current legal framework already provided stimulation for rare diseases.
*The Dutch Ministry of Public Health, Welfare and Sports, Drugs and Medical Technology considered a EU regulation in this matter not the right way forward as there are different reimbursement systems within the different EU Member States.
*Baxter Healthcare did not see any justification to introduce such legislation.

Of all the overwhelmingly positive reactions, I cite only a few here:

*The Finnish rare-disease patient organization replied to this question of the consultation that developing equipment and determining norms in the EU would help those countries who still have challenges to improve their national standards. The ability to improve the national standard was considered insufficient in many EU countries and the markets too small.
*The Swedish government agreed that there was a need to investigate the conditions required for developing incentive measures and legislation for orphan devices similar to orphan drugs, but they suggested a thorough analysis of the financial impact and possible rules should first take place.
*The UK Genetic Interest Group (patients) suggested that the burden of regulation should be kept to a minimum with a single European application.

Nevertheless, “orphan-device” legislation has yet to be introduced in Europe today [7] as we have for orphan medicinal products since 2000. Only some EU Member States have national rules (for Belgium: Royal Decrees 15 July 1997 and 18 March 1999) for medical devices (“*dispositif à usage unique*”).

In the United States of America a Humanitarian Use Device (HUD) [8, 9] is a device that is intended to benefit patients by treating or diagnosing a disease or condition that affects or is manifested in fewer than 4000 individuals in the United States per year. The application (since 1990) must contain sufficient information for the FDA to determine that the device does not pose an unreasonable or significant risk of illness or injury and that the probable benefit to health outweighs the risk of injury or illness from its use taking into account the probable risks and benefits of currently available devices or alternative forms of treatment. Up to the present, 65 HUD’s have been approved by FDA, mostly implantable (programmable) therapeutic devices in pediatrics (pediatric devices) [10], cardiology (ventricular assist devices in congestive heart failure) [11], neurology (microelectrodes for neurostimulation), hematology (cryofilter for cryofiltration apheresis) [12], otorhinolaryngology (auditory brain stem implants) and orthopedics (craniosynostosis).

Custom made (active implantable) medical devices are devices that are made by special request (eventually 3D-printed) of a health professional intended to be used for a particular patient [13]. Several agencies (UK, TGA, FDA) [14] have regulations in place to allow the use of these unique one-time devices foremost in unmet medical need situations. Technological changes, improvements and refinements provide a much shorter marketing period to obtain revenue to develop newer versions of the device.

### Position: policy recommendation

A regulatory European framework with economic incentives needs to be installed to stimulate the research and development of orphan devices similar to the legislation around orphan drugs. Incentives are needed (as for orphan drugs) to enable useful medical devices to reach the patients and clinicians in a timely fashion. Collection and analysis of publicly accessible safety/efficacy data (EUDAMED: European Databank on Medical Devices) needs to be centralized between all EU Member States to reach a sufficient number of patients to perform comparative-(cost)effectiveness and –safety studies.

All pictures are taken from the archives of HistArUz, the Museum of the History of Medicine and Pharmacy at the University Hospitals Leuven, Belgium: http://www.uzleuven.be/histaruz.
